# Research on the performance of phase change energy storage devices based on solar energy

**DOI:** 10.1371/journal.pone.0322491

**Published:** 2025-04-28

**Authors:** Ningge Xu, Chunlong Zhuang, Guangqin Huang, Hongyu Zhang, Fei Gan, Shanshan Hou, Lei Cheng, Yi Huang

**Affiliations:** Facilities Department, Army Logistics Academy, Chongqing, China; Southwest Jiaotong University, CHINA

## Abstract

This article designs a high-altitude border guard post that can fully utilize the heat absorbed by solar collectors to continuously store thermal energy during the day and stably release heat at night. This device is a spherical encapsulated paraffin phase change heat exchanger device (stainless steel shell diameter: 80mm),By conducting thermal storage and release experiments on the device, the performance of the device was analyzed. The experimental results showed that in the thermal storage experiment, the outlet temperature of the hot fluid reached about 97 °C at about 500 minutes and tended to stabilize. The wall temperature and center temperature of the encapsulated paraffin sphere began to stabilize at about 450 minutes and stopped absorbing heat; In the heat release experiment, the wall temperature and core temperature of the encapsulated paraffin spheres stabilized at about 400 minutes and stopped releasing heat; In this way, it can meet the heating needs of storing heat for about 8 hours during the day and continuously releasing heat for 6.5 hours at night.

## 1 Introduction

The highland and alpine border post area is known as “no birds in the sky, no grass on the ground, wearing cotton jackets all year round, not enough oxygen to eat, sunburned skin, wind-blown stone running.” The natural environment is extremely harsh. The natural environment is extremely harsh, characterized by very cold temperatures but very rich solar energy resources. The area where the border post is located (in Tibet, for example) in winter has the lowest temperature down to −40 °C, part of the annual average temperature below −10 °C. However, the daytime sunshine in such areas is very cold but very rich in solar energy resources. However, these areas are very sunny during the day and have long hours of sunshine. Still take Tibet as an example. Tibet is China and even the world’s richest region of sunlight energy in a province. Solar energy resources are very rich, in which the most abundant solar irradiation in the region of direct solar radiation is in the 6700 ~ 8370 MJ/m^2^ per year, equivalent to 230 kg standard coal combustion of the heat emitted by the [1]. At present, the heating in the border guard post area mainly relies on coal-fired heating, and most of the coal needs to be transported from the mainland by highway and railroad, and it is difficult to raise all kinds of fuels locally, so the traffic and road conditions in the area have a direct impact on the guarantee of heating. In terms of heating methods, centralized heating is not suitable for decentralized barracks and field stations, and the use of independent heating facilities is not energy-efficient, resulting in high operating costs. When there is a lack of energy supply, heating in these areas often relies on firewood or cow dung as fuel, which completely fails to meet the requirements of the construction of energy-saving and ecological camps. Based on the sunshine conditions in such areas, solar energy has become an ideal renewable energy source to be utilized, and solar thermal conversion technology has become a major initiative for sustainable development. Therefore, in China’s highland alpine post areas, the use of solar heating is a very effective and convenient method.

Solar thermal energy storage technology is categorized into sensible heat storage, latent heat storage, and chemical reaction heat storage according to the thermal energy storage method [2].(1) The sensible heat storage system utilizes the specific heat capacity of the material and the temperature change of the material in the process of energy storage and energy release. The specific heat capacity of the material is approximately constant over a small temperature range, so the greatest advantage of sensible heat energy storage is that the energy storage and release process is completely reversible over the effective service life of the system. Moreover, during the operation of the system, there are fewer instability factors to be considered technically, so the sensible heat energy storage system is simple in structure and easy to operate. However, its most important disadvantage is that its energy storage density is small, that is, the energy that can be stored per unit volume is small, and the volume of the energy storage device is often too large [3].(2) Latent heat energy storage is the use of material in the solidification, melting, condensation, gasification, sublimation, and other forms of phase change processes that are to be absorbed or released as latent heat of the phase change principle of heat storage, so it can also be called phase change energy storage. From the point of view of energy density, latent heat storage is much larger than the heat stored in the sensible heat [4].(3) Chemical reaction energy storage is to use the reaction heat of a reversible chemical reaction to store energy; this way of energy storage density is larger, but the technology is complex and inconvenient to use. Therefore, the storage capacity of phase change energy storage is higher than sensible heat energy storage, and the technology is simpler than chemical reaction energy storage.

Phase change storage technology attracts a lot of research on it by virtue of its superiority, and the development momentum is strong. Phase change energy storage technology is based on phase change energy storage materials as the basis of high technology, phase change materials Phase change latent heat is large, much larger than the apparent heat energy storage density. In the process of storing and releasing energy, the material occurs in isothermal or nearly isothermal conditions, the temperature and thermal energy are basically stable, and they can be used as a basis for the development of industrial energy-saving system systems and high-tech products to meet the people of the system and the products’ special performance and cost requirements [5–8].

For the research of phase change materials since the 1970s, continuous and systematic research has been carried out at home and abroad on traditional phase change materials such as inorganic salts, inorganic water-containing salts, and metals. The Tibet Solar Energy Research and Demonstration Center, in cooperation with Central China Normal University, has successfully developed solar energy high energy storage density phase change materials by mixing inorganic water-containing salt materials such as manganese nitrate and borax with nucleating agents in moderate proportions. Currently, commonly used phase change materials include organic materials represented by fatty acids and paraffins and inorganic materials represented by hydrated salts. Paraffin-based phase change materials are stable, non-corrosive, and readily available phase change energy storage materials. Li Xinguo et al. [9] established an experimental system of phase change in a circular outer tube using paraffin wax as phase change material and obtained the phase change law of paraffin wax at different positions during heat storage and exothermic storage as well as the melting time of paraffin wax. Yang Jialin et al. [10] The phase change temperature and latent heat of phase change of paraffin were obtained by measuring the thermophysical properties of paraffin by DSC oscillometric scanning calorimetry using a shell-and-tube phase change heat storage structure as an object. It provides a certain basis for selecting phase change materials with a better heat exchange effect.

A phase change heat accumulator (PCHA) functions as a specialized heat exchanger where thermal performance is optimized by enhancing three key parameters in the heat transfer equation：Q = K·A·∆T (Where Q is the heat transfer rate (W), K is the overall heat transfer coefficient (W/m²·K), A the effective heat transfer area (m²), and ΔT is the temperature gradient (K)). It can be seen that increasing the integrated heat transfer coefficient, increasing the effective heat transfer area, and increasing the difference in temperature of the heat transfer can increase the amount of heat transfer [11]. Decentralized encapsulation of phase change materials is also relatively common in practical applications. Decentralized encapsulation is to use metal, plastic, and other materials made of tubes, balls, plates, and other shapes of the shell, and the phase change material is encapsulated in it, which is most widely used in the form of spherical shell encapsulation. The application is generally more than one phase change material encapsulation body stacked as a bed-type heat storage and through the heat transfer fluid and the outside world for heat transfer, so that the heat storage involves more than one encapsulation body inside the phase change material phase change heat transfer, but also its convection heat transfer with the heat transfer fluid and heat conduction, which is a complex non-steady state heat transfer process. In this article, paraffin of the device with 58.5–62.5°C phase transition temperature maximizes ΔT with 112°C solar heat sources, achieving 41% higher daily storage than those of hydrated salts, 80mm-diameter stainless steel spheres provide 92% packing density and survive -40°C thermal stress, reducing annual efficiency decay to <3%, Foldable baffle design self-adjusts airflow for 27–33 kPa ambient pressure, maintaining 1.45 m/s flow rate without auxiliary power.

## 2 Experimental section

### 2.1 Experimental setup

#### 2.1.1 Solar phase change energy storage devices.

**Fig 1:**
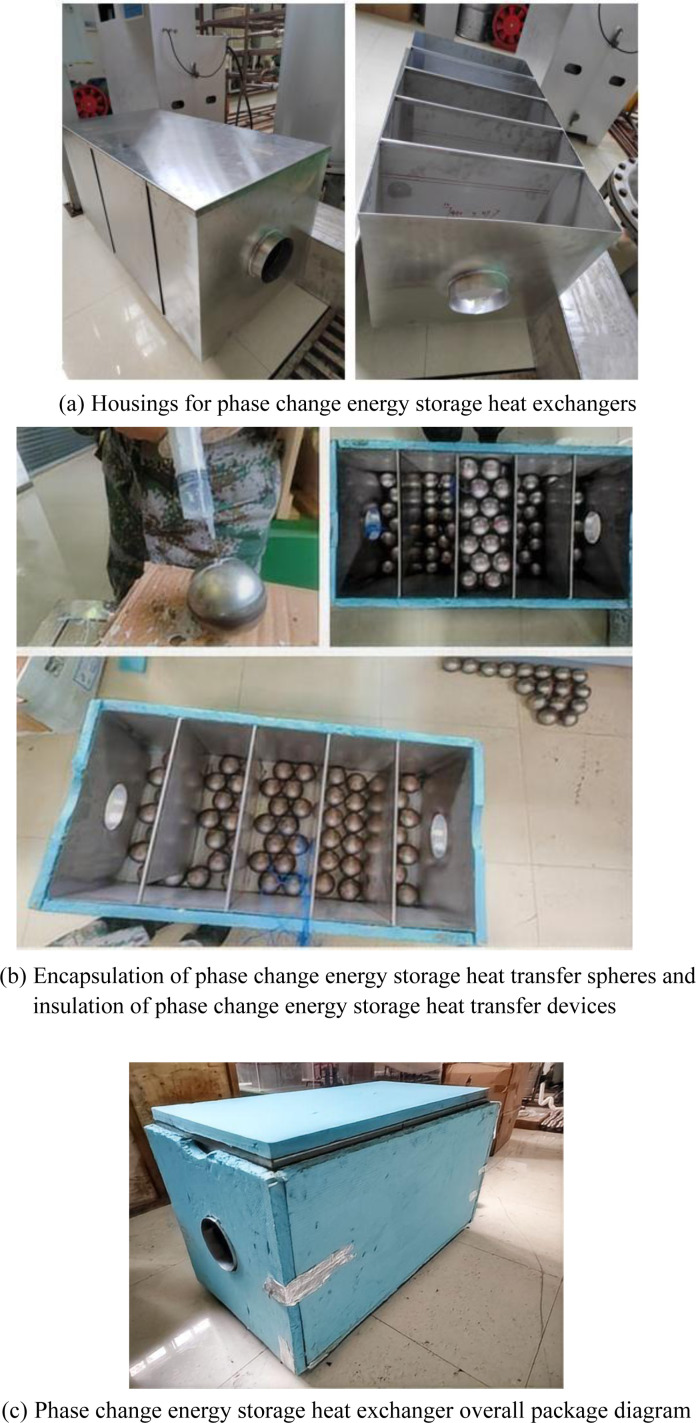
Schematic of Spherical PCM Encapsulation and Solar-TES System Integration.

As shown in Fig 1.The solar phase change energy storage and heat transfer device is composed of stainless steel with an external dimension of 1500*1000 mm, and 471 phase change spheres are loaded inside; the phase change spheres are filled with paraffin waxes with a phase change temperature of 58.5~62.5°C by the stainless steel shell with a diameter of 80mm (the physical parameters are shown in Table 1), and the shell of the 471 spheres is filled with about 85 kg of paraffin waxes; the device is opened with ventilation openings with a diameter of 150mm (one inlet and one outlet) in front of and behind the device. The diameter of the device is 150 mm (an inlet and an outlet); its internal folding plate can be disassembled at any time according to the experimental needs; the device’s box is outside the use of high-density XPS extruded plastic board as a thermal insulation layer.The design of the spherical encapsulation method used in this study is supported by previous research on the optimization of heat transfer in PCM-based systems. Hosseini et al. investigated the effect of geometric and operating parameters on the thermal behavior of shell-and-tube latent heat energy storage systems, providing insights into the design of efficient heat exchangers [12]. Additionally, Hua et al. explored the use of nano-metal/paraffin composite PCMs, demonstrating enhanced thermal conductivity and heat transfer performance [13].

**Table 1:** Thermophysical Properties of Paraffin Phase Change Material (PCM).

Phase transition temperature/K	latent heat of phase transition /(kJ- kg)^-1^	Density/(kg·m)^-3^		Thermal conductivity/(W· (m· K))^-1^		Specific heat capacity/(kJ· (kg· K))^-1^	
**solid state (physics)**	**liquid (state)**	**solid state (physics)**	**liquid (state)**	**solid state (physics)**	**liquid (state)**
335.65	248.95	916	776	0.28	0.14	1.7	2.5

#### 2.1.2 Physical parameters of the material.

The phase change material used in this experiment is paraffin wax and the physical parameters are shown in Table 1, which will be used in the analysis of the experimental results.

#### 2.1.3 Overall experimental setup.

**Fig 2:**
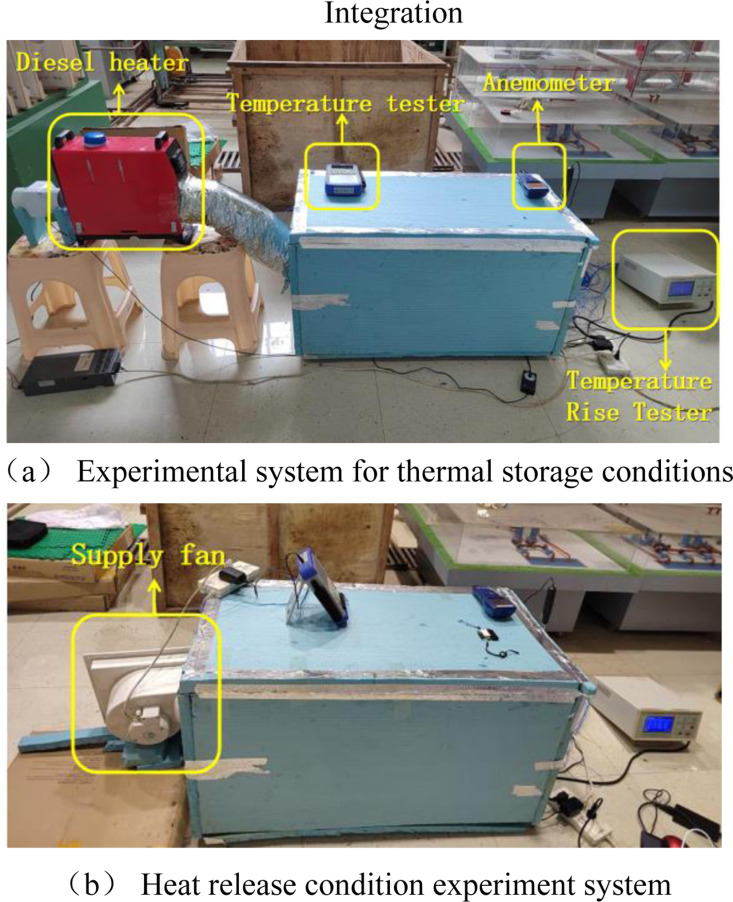
Diagram of experimental setup.

The experimental setup is shown in Fig 2, and the functions of each part of the setup are shown as labeled in the figure, where the heating supply fan simulates the temperature and speed of the air coming out of the solar collector.

### 2.2 Experimental process

#### 2.2.1 Test conditions.

The experiment is divided into two parts: heat storage experiment and heat release experiment. In the heat storage experiment, the air inlet temperature varied little, with a difference of 6.4°C between the maximum and minimum values, and the average value was 112.03°C, and the flow rate at the outlet was about 1.45 m/s. In the heat release experiment, the air inlet temperature was about 27.2°C, and the flow rate at the outlet was 2.9 m/s.The operating conditions and physical properties for heat storage and release are listed in Table 2.

**Table 2:** Operating Conditions and Thermophysical Properties for Heat Storage/Release Experiments.

Parameter	Heat Storage Condition	Heat Release Condition
Inlet temperature (°C)	112.03	27.2
Outletvelocity (ms^-1^)	1.45	2.9
**PCM Properties**		
Phase Change Temp. (°C)	58.5–62.5	58.5–62.5
Latent Heat (kJ/kg)	248.95	248.95
Solid Density (kgm^-^³)	916	916
Liquid Density (kgm^-^³)	776	776
**HTF Properties**		
Fluid Type	Air	Air
Specific Heat (kJ/kg·K)	1.005	1.005
Density (kgm^-^³)	0.908	1.177
Thermal Conductivity (W/m·K)	0.032	0.026

#### 2.2.2 Test equipment.

To ensure transparency and reproducibility, Table 3 summarizes the technical specifications of all critical instruments used in the experimental setup. The selection criteria for these devices prioritized measurement accuracy (±0.5°C for temperature, ±2% for airflow), compatibility with alpine operating conditions (-50–260°C temperature resistance), and alignment with the system’s thermal power range (2000–5000 W). Detailed parameters—including sensor types, power requirements, and operational ranges—are provided to facilitate replication of the heat storage/release experiments.

**Table 3:** Specifications of Experimental Equipment.

	Device	Model	Key Parameters	Power Supply	Sensor/Probe Type
1	Multi-Circuit Temperature Tester	JK500C (Kinko)	Range: -200~1300°C Scanning speed: 100 ms ~1 s Accuracy: ±0.5°C	100–240 V AC, 50/60 Hz, 0.35 A	NiCr-NiSi (K-type thermocouple)
2	Multi-Circuit Temperature Rise Tester	SH-X (Shenhua)	Range: -100~1000°C Accuracy: ±0.5°C	220 V AC ±10%, 50 Hz ±2%	NiCr-NiSi (K-type thermocouple)
3	Temperature/Humidity/Wind Speed Meter	TSI-9545	Wind speed: 0~30 m/s Humidity: 0~95% RH	N/A (Battery-powered)	Integrated thermal anemometer
4	K-type PTFE Thermocouple Wire	N/A	Insulation: PTFE- Wire diameter: 2 mm × 0.3 mm Temp. resistance: -50~260°C	N/A	Nickel-chromium/nickel-silicon
5	Diesel heater	5000W	fuel: diesel; fuel consumption: 0.23~0.51 L/H; power consumption: 13~42W; air flow:120~190m^3^/h	2000~5000W	

#### 2.2.3 Measurement point layout.

**Fig 3:**
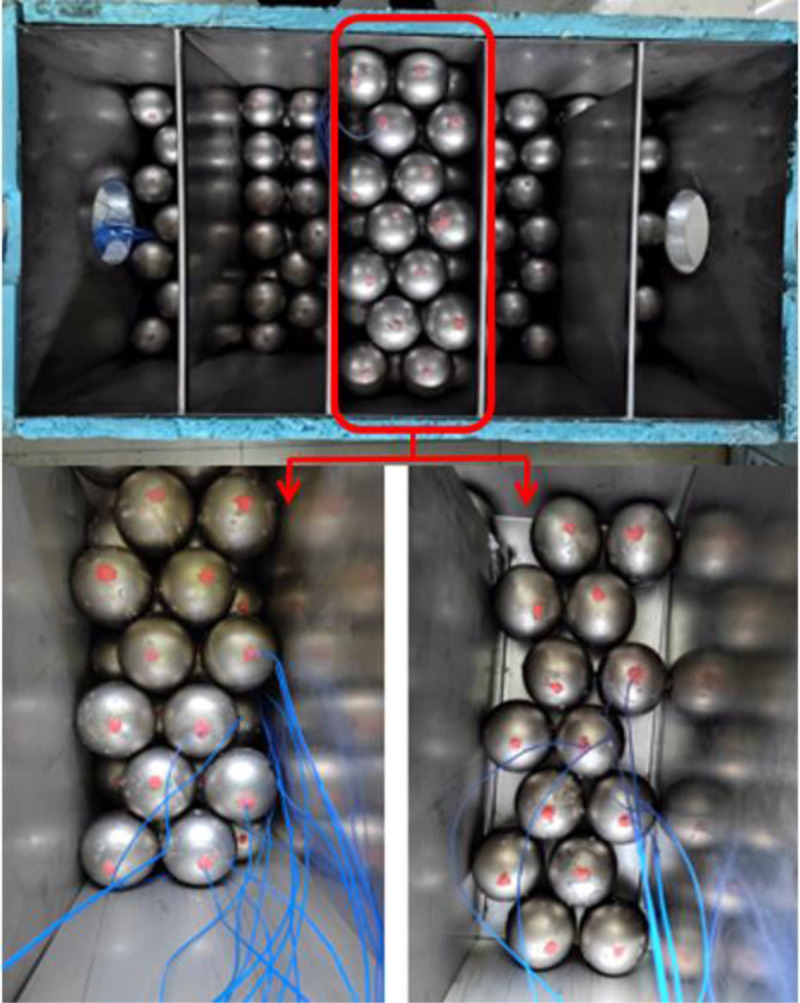
Arrangement of temperature measurement points of solar phase change energy storage heat exchanger device.

**Fig 4:**
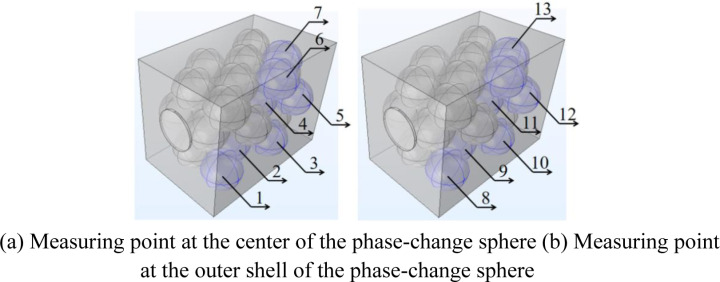
Temperature measurement point markers for local simulation of solar phase change energy storage heat exchanger unit.

A total of 15 measurement points are arranged in the solar phase change energy storage heat exchanger, which are divided into three parts: 7 temperature measurement points are arranged at the center of the phase change sphere, marked as points 1–7; 6 temperature measurement points are arranged on the outer wall of the phase change sphere, and the temperature line at each point is arranged perpendicularly to the ground, marked as points 8–13; and 2 measurement points are arranged in the entrance and exit of the box, marked as points 14 and 15. As the distribution of the device is an axisymmetric structure, so the third grid in the middle position (folding plate will be the Yangneng phase-change energy storage and heat transfer device is separated into five compartments) as the object of the temperature measurement point layout, Fig 3 is the solar energy phase-change energy storage and heat transfer device in-kind diagram of the temperature measurement point layout; and the third grid of the phase-change sphere is also placed in the position of the axis of symmetry, so in order to facilitate the marking of the construction of the third grid of the local schematic diagram will be The temperature measurement points of the physical figure Fig 3 are marked on the simulation figure Fig 4.

## 3 Experimental results and discussion

### 3.1 Thermal storage experiment results and discussion

**Fig 5:**
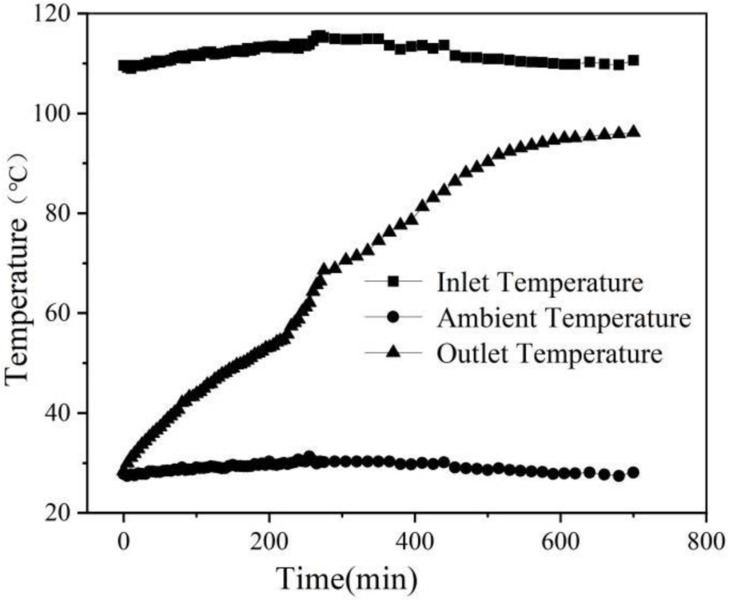
Inlet and Outlet Air Temperature Profiles During Thermal Storage (Hot Fluid: 112°C).

In this experiment, the air temperature at the inlet and outlet of the phase change energy storage device was measured. In the thermal storage experiment, it can be seen from Fig 5 that the inlet temperature always stays within the range of 110~112.7°C, which indicates that the inlet temperature is relatively stable and is little affected by the ambient temperature. While the export temperature increases with time, after 500 min or so, it is stable. This is due to the phase change sphere temperature being lower than the temperature of the air flowing over its surface during this period of time. The hot air is constantly exothermic to the phase change sphere, so that the temperature of the phase change sphere is gradually increased. In the case of small changes in the import temperature, the heat transfer temperature difference between the air and the phase change sphere gradually decreases, and the phase change sphere gradually reduces the amount of heat absorption, resulting in a gradual increase in the air outlet temperature.

The curve in the figure in 230 ~ 300 min appeared in a period of steep rise. This is due to the phase change sphere in this period of time phase change; the sphere inside the proportion of solid phase gradually decreased, the proportion of liquid phase is increasing, and the phase change material paraffin wax in the liquid state of thermal conductivity is only 1/2 of the solid state; that is to say, with the increase of the proportion of liquid in the phase change sphere body, the heat absorption of the phase change sphere is reduced, resulting in the reduction of air exothermic, and the outlet air temperature rises rapidly.

As can be seen from the figure, after 500 minutes, the outlet air temperature of the device tends to stabilize, indicating that the phase-change sphere no longer absorbs heat and the heat storage is completed. It can be seen that the phase change energy storage device can be completed in about 8 hours of heat storage, and daytime sunshine time fits. After 8 hours of heat storage, the temperature difference between the air import and export is basically unchanged, about 14.4 °C, which is caused by the heat loss of the heat storage box. In the late stage of heat storage, the surface temperature of the heat storage box reaches more than 40 °C, while the ambient temperature is about 27.2 °C, which has a large temperature difference in the heat transfer, resulting in a certain amount of heat loss.

**Fig 6:**
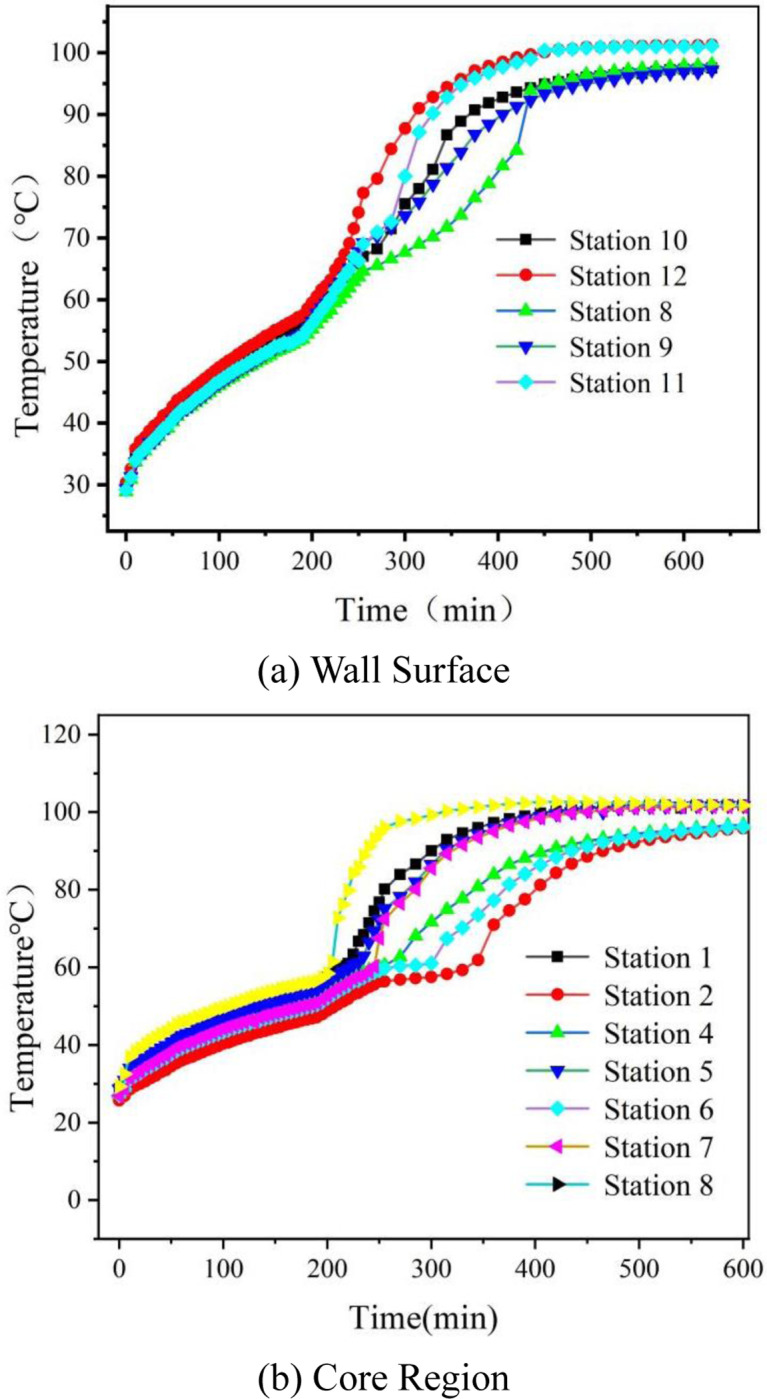
Transient Temperature Distribution in PCM Spheres During Thermal Storage: (a) Wall Surface, (b) Core Region.

The temperature drop (ΔT_storage_=T_in_−T_out_) quantifies heat transfer efficiency from air to PCM. ΔT_storage_ peaks at 84.3°C (112°C inlet vs. 27.7°C outlet at t = 50 min) and stabilizes to 14.4°C after 500 min as PCM saturates. This aligns with the phase change completion observed in Fig 6.

Fig 6 illustrate the temperature changes over time for both the center and wall temperatures of the phase change sphere during the heat storage experiment. It can be observed from the figures that, despite the different positions of the phase change spheres, the trends in temperature change over time are consistent. Initially, both the wall temperature and the center temperature of the sphere increase with time, although the rate of increase is slow. Around 200 minutes, both graphs exhibit a distinct inflection point, at which the temperature reaches approximately 59 °C. Following this point, the temperature rises sharply, indicating that the phase change material is undergoing a phase change. After the phase change, the increase in sensible heat causes a significant rise in temperature. By approximately 450 minutes, the temperature of the phase change sphere approaches that of the hot air, resulting in the cessation of heat exchange.The 450 min stabilization time is governed by paraffin’s low liquid-phase conductivity (k_liquid_=0.14 W/m·K), which throttles heat transfer during phase change.

### 3.2 Heat release experiment results and discussion

**Fig 7:**
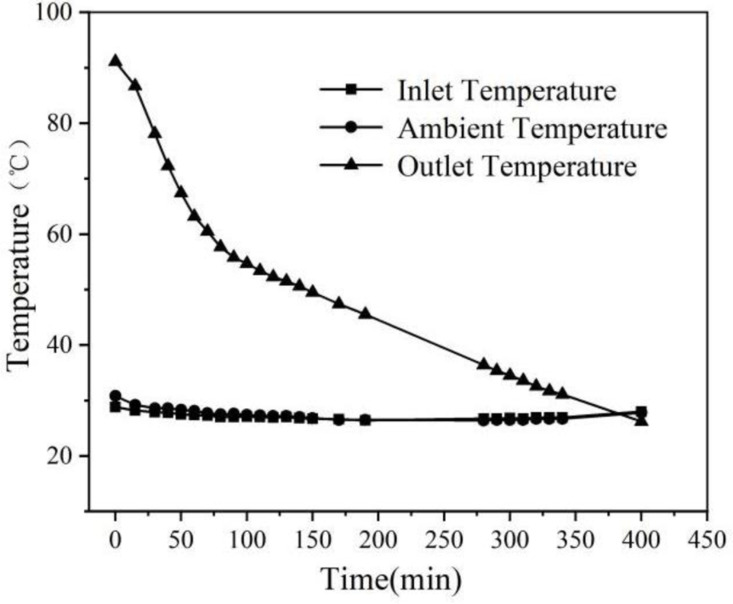
Heat release experiment inlet and outlet air temperature variation with time.

Fig 7 illustrates the relationship between the import and export air temperatures over time during the heat release experiment. As depicted in the figure, at the initial moment of the experiment, the imported air temperature is significantly lower than the temperature of the phase change sphere. This creates a substantial temperature difference, causing the heat storage ball to release a large amount of heat to the air, resulting in a higher export temperature of approximately 92 °C. However, as the heat storage ball continues to release heat, its temperature gradually decreases. Consequently, the heat transfer between the air and the sphere diminishes, leading to a continuous decline in the export air temperature, indicating that the heat release from the device has been completed.

**Fig 8:**
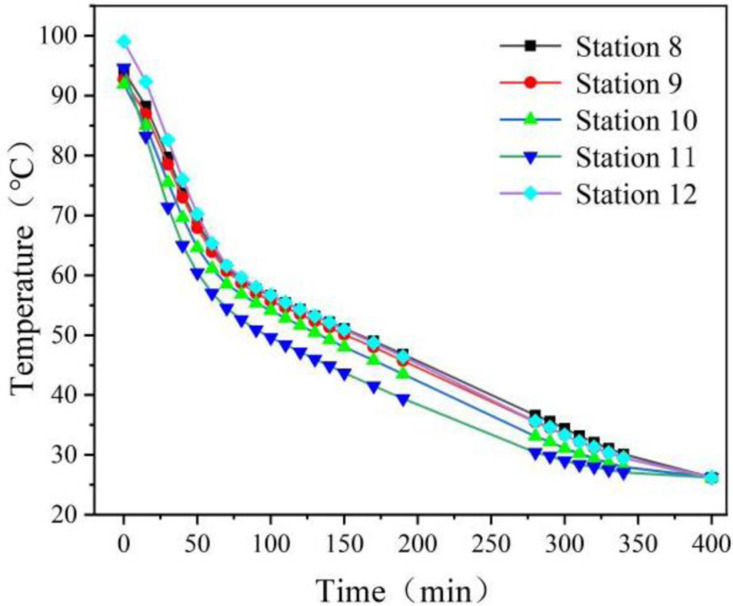
Wall temperature versus time for phase change spheres in heat release experiments.

**Fig 9:**
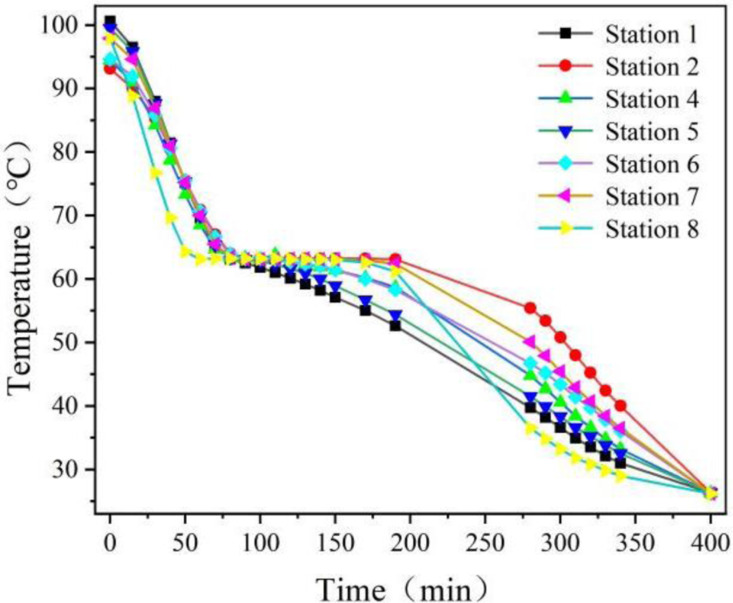
Plot of sphere center temperature versus time for phase change spheres in heat release experiments.

Fig 8 and 9 illustrate the curves representing the wall temperature and the center temperature of the phase change sphere over time during the heat release experiment. Between 60 and 75 minutes, both figures exhibit distinct inflection points, particularly in Fig 9. Following the appearance of the inflection point, the temperature stabilizes, indicating that during this period, the phase-change material begins its phase transition. Approximately 400 minutes into the experiment, all temperatures converge to nearly the same value, around 26°C. This suggests that, at this point, the heat release from the device to the surrounding air has concluded.

It can be observed that the device is capable of continuously releasing heat into the air for approximately 6.5 hours, which meets the requirement for heat release during the night. The 6.5 hour heat release duration is enabled by turbulent airflow (Re = 2900), achieving a Nusselt number Nu=18.3 for efficient heat extraction.

### 3.3 Thermal power of the device

**Fig 10:**
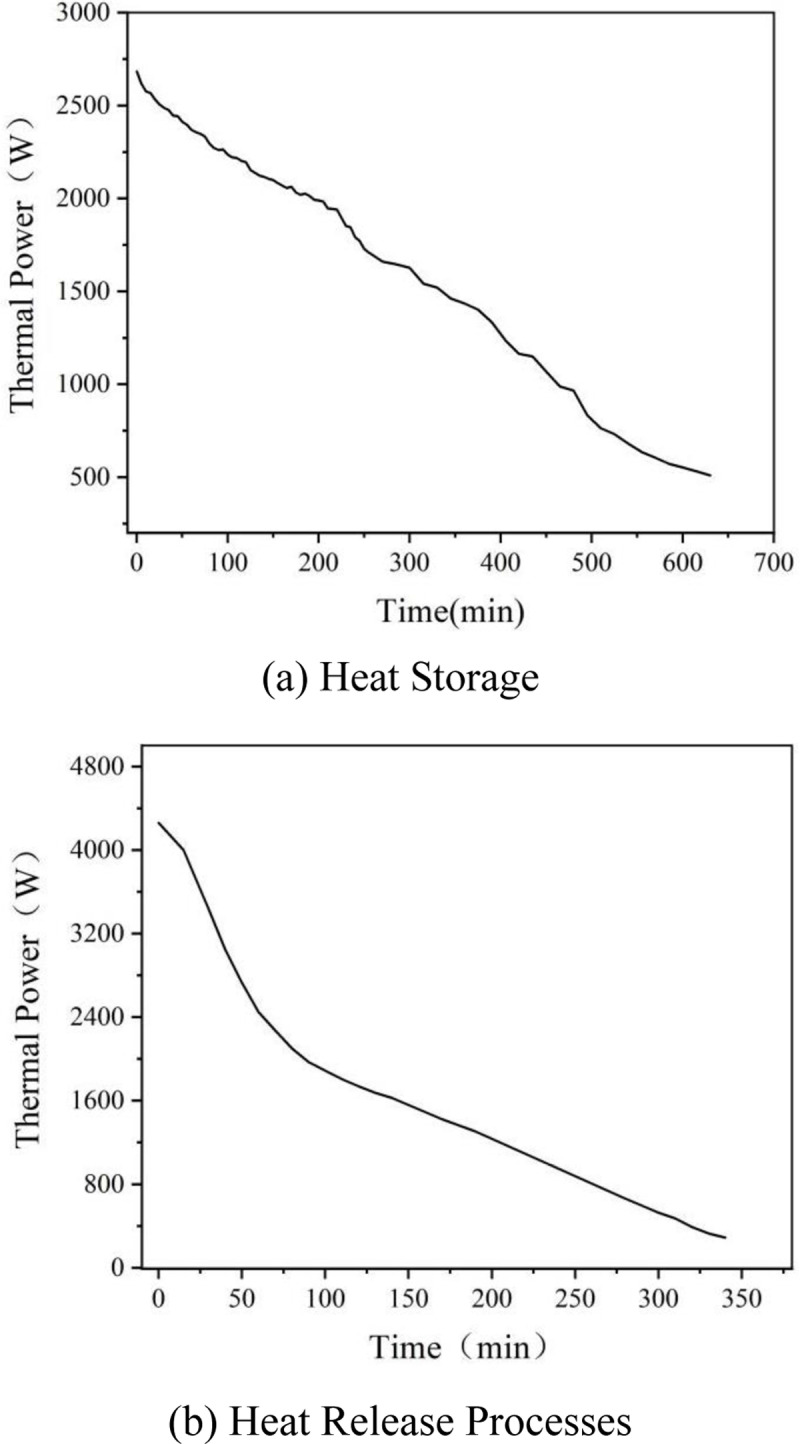
Thermal Power Decay During (a) Heat Storage and (b) Heat Release Processes.

According to the experimental data presented, the thermal power variation over time for both the heat storage and heat release experiments has been calculated, as illustrated in Fig 10 The figures indicate that the thermal power in both the heat storage and heat release processes decreases over time; however, the rate of decrease in the heat storage process is greater than that in the heat release process. This discrepancy can be attributed to the higher heat loss experienced during the heat storage process, which occurs due to a significant temperature difference between the thermal fluid and the surrounding environment. Consequently, the heat transfer efficiency in the heat release process is superior to that in the heat storage process.

## 4 Conclusion

Aiming to address the characteristics of prolonged sunshine duration and low average daily temperatures in alpine regions, we designed and constructed a solar energy phase-change energy storage device. This device facilitates heat storage during the day and releases heat at night through the use of paraffin wax as the phase-change material. The goal is to provide heating to outposts during nighttime.

The performance study of the device, including storage and exothermic experiments, yielded the following results: In the heat storage experiment, the temperatures of the hot air outlet, phase-change sphere core, and wall continued to rise over time, stabilizing between 450 and 500 minutes. This indicates that the device has completed its heat storage phase. In the exothermic experiments, the temperatures of the cold air outlet, phase-change sphere core, and wall decreased over time, stabilizing between 380 and 400 minutes, which indicates that the device has ceased exothermic activity. It can be concluded that this phase-change energy storage device is capable of achieving heat storage during the day for 8 hours and exothermic release at night for approximately 6.5 hours. This performance is sufficient to meet the heating demands of the guard post during nighttime.
